# High-Efficiency Electromagnetic Interference Shielding of rGO@FeNi/Epoxy Composites with Regular Honeycomb Structures

**DOI:** 10.1007/s40820-022-00798-5

**Published:** 2022-01-27

**Authors:** Ping Song, Zhonglei Ma, Hua Qiu, Yifan Ru, Junwei Gu

**Affiliations:** 1 Shaanxi Key Laboratory of Macromolecular Science and Technology, School of Chemistry and Chemical Engineering, Northwestern Polytechnical University, Xi’an, 710072 People’s Republic of China; 2 Queen Mary University of London Engineering School, Northwestern Polytechnical University, Xi’an, 710072 People’s Republic of China

**Keywords:** Electromagnetic interference shielding, rGO@FeNi, Epoxy resins, Honeycomb structures

## Abstract

With the rapid development of fifth-generation mobile communication technology and wearable electronic devices, electromagnetic interference and radiation pollution caused by electromagnetic waves have attracted worldwide attention. Therefore, the design and development of highly efficient EMI shielding materials are of great importance. In this work, the three-dimensional graphene oxide (GO) with regular honeycomb structure (GH) is firstly constructed by sacrificial template and freeze-drying methods. Then, the amino functionalized FeNi alloy particles (*f*-FeNi) are loaded on the GH skeleton followed by in-situ reduction to prepare rGH@FeNi aerogel. Finally, the rGH@FeNi/epoxy EMI shielding composites with regular honeycomb structure is obtained by vacuum-assisted impregnation of epoxy resin. Benefitting from the construction of regular honeycomb structure and electromagnetic synergistic effect, the rGH@FeNi/epoxy composites with a low rGH@FeNi mass fraction of 2.1 wt% (rGH and *f*-FeNi are 1.2 and 0.9 wt%, respectively) exhibit a high EMI shielding effectiveness (EMI SE) of 46 dB, which is 5.8 times of that (8 dB) for rGO/FeNi/epoxy composites with the same rGO/FeNi mass fraction. At the same time, the rGH@FeNi/epoxy composites also possess excellent thermal stability (heat-resistance index and temperature at the maximum decomposition rate are 179.1 and 389.0 °C respectively) and mechanical properties (storage modulus is 8296.2 MPa).

**Supplementary Information:**

The online version contains supplementary material available at 10.1007/s40820-022-00798-5.

## Highlights

- The rGH@FeNi/epoxy electromagnetic interference (EMI) shielding composites with regular 3D honeycomb structures were prepared by sacrificial template, freeze-drying and vacuum-assisted impregnation of epoxy resin.
- The construction of 3D honeycomb structure and electromagnetic synergistic effect significantly increase the EMI shielding effectiveness and reduce the secondary contamination.
- The rGH@FeNi/epoxy composites possess excellent thermal stability and mechanical properties.

**Supplementary Information:**

The online version contains supplementary material available at 10.1007/s40820-022-00798-5.

## Graphical abstract

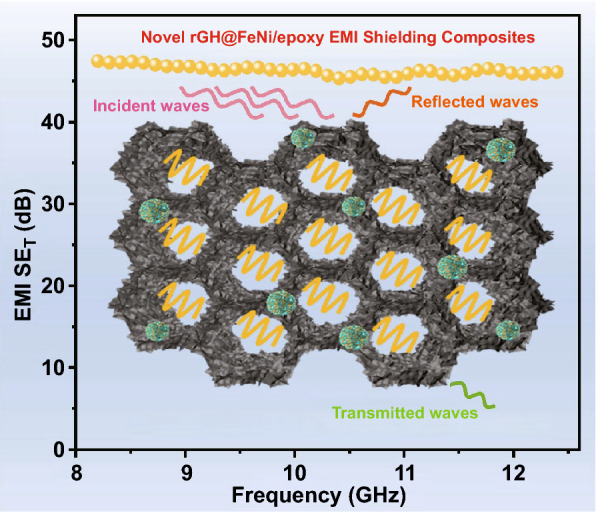

## Introduction

With the rapid development of fifth-generation (5G) communication technology and wearable electronic devices, electromagnetic interference (EMI) and radiation pollution caused by electromagnetic waves have become prominent issues in consumer electronics, artificial intelligence and information security, etc. The leakage of electromagnetic waves will not only cause malfunctioning issues of electronic communication equipment and its delicate components, but also pose serious threats to human health [1–4]. Polymer-based EMI shielding composites have become an important research direction of efficient EMI shielding materials due to their advantages of light weight, corrosion resistance, stable EMI shielding performance and outstanding mechanical properties [5–7].

Currently, polymer-based EMI shielding composites mainly achieve their excellent EMI shielding performances by incorporating conductive and/or magnetic functional fillers into polymer matrix. Commonly used polymer matrixes include natural rubber (NR), polyethylene (PE), polypropylene (PP), polyurethane (PU), polyvinylidene fluoride (PVDF), polyimide (PI) and epoxy resins, etc*.* [8–10]. The conductive fillers usually include metals, conductive polymers and carbon materials. Among them, carbon materials such as carbon black, graphite, carbon fiber, carbon nanotubes (CNTs) and reduced graphene oxide (rGO) have attracted extensive attention due to their advantages of low density, high electrical conductivity (*σ*), corrosion resistance and easy processing [11–13]. The rGO, especially, has been widely used in EMI shielding due to its unique two-dimensional lamellar structure, excellent *σ* and outstanding mechanical properties [14–16]. However, rGO tends to agglomerate in polymer matrix and has a large contact resistance, resulting in the relatively high conductive percolation threshold of polymer-based composites [17, 18]. Therefore, high rGO mass fraction is usually required to obtain excellent EMI shielding performance, which greatly affects the mechanical properties and processabilities of the composites [19].

The construction of efficient three-dimensional (3D) conductive networks can effectively reduce the percolation threshold of rGO/polymer composites, so as to realize the synergistic improvement of *σ*, EMI shielding performances and mechanical properties for polymer composites at low filler content [20, 21]. Current methods for constructing efficient 3D conductive networks in polymer-based composites mainly include foaming, surface coating-hot pressing and sol–gel methods, etc*.* [22]. Eswaraiah et al. [23] reported the fabrication of functionalized rGO/PVDF foam composites using 2,2’-azobiso-butanitrile (AIBN) as the chemical foaming agent. The resultant rGO/PVDF foam composites had an EMI shielding effectiveness (EMI SE) of 28 dB when the rGO mass fraction was 7 wt%. Yan et al. [24] fabricated the rGO/PS EMI shielding composites with segregated structure by dip-coating rGO onto the surface of PS particles followed by hot pressing them together. The EMI SE of rGO/PS composites reached 45 dB when the rGO volume fraction was 3.47 vol%. In our previous work, Gu et al. [25] firstly prepared the graphite nanoplatelets (GNPs)/rGO aerogel by sol–gel method, and then obtained the GNPs/rGO/epoxy EMI shielding composites by vacuum-assisted impregnation of epoxy resin. The results show that the EMI SE of GNPs/rGO/epoxy nanocomposites reached 51 dB when the rGO and GNPs mass fractions were 0.1 and 20.4 wt%, respectively.

Although the constructed 3D conductive networks can endow the polymer composites with excellent *σ* and EMI shielding performances at low filler content, the conductive networks obtained by the above methods have poor structural regularity and the distribution of nanofillers is not easy to control, leading to the poor stability of *σ* and EMI shielding performances [26, 27]. The rGO conductive networks with honeycomb structure based on alumina (Al_2_O_3_) honeycomb plates possess the advantages of regular morphology, and controllable size, shape and distribution of holes, as well as good repeatability and stability. In our previous work, Gu et al. [28] used Al_2_O_3_ honeycomb plates as templates to prepare the rGO with regular honeycomb structure (rGH)/epoxy EMI shielding composites by sacrificial template, freeze-drying and vacuum-impregnation methods. The obtained rGH/epoxy composites at a mass fraction of 1.2 wt% rGH exhibit an elevated EMI SE value of 38 dB, which was 6.3 times of that of the homogeneously blended rGO/epoxy composites at the same rGO mass fraction.

In addition, the EMI shielding mechanism of the existing polymer-based composites is mainly based on conduction loss, and the impedance mismatch between their surface and air results in the reflections of electromagnetic waves on the surface of composites, which is easy to cause the secondary electromagnetic wave pollution [29, 30]. Studies have shown that the introduction of magnetic materials can improve the impedance match between the composites and external space, thus reducing the reflections of electromagnetic waves and secondary contamination [31–33]. The magnetic materials mainly include pure metal (iron, cobalt and nickel, etc*.*), metal oxides (iron oxide, ferrite and nickel oxide, etc*.*) and metal alloys (iron-cobalt alloy, iron-nickel alloy and cobalt–nickel alloy, etc*.*). FeNi alloy exhibits excellent initial permeability, relative permeability, as well as low coercivity and repeated magnetization loss, and shows great potentials in EMI shielding applications [34–36].

In this paper, Al_2_O_3_ honeycomb plates were used as templates to construct GO with regular honeycomb structure (GH) by sacrificial template and freeze-drying. Then, the surface functionalized FeNi alloy particles (*f*-FeNi) were loaded on the GH aerogel and reduced in situ to prepare magnetic and conductive rGH@FeNi aerogel Finally, rGH@FeNi/epoxy EMI shielding composites with regular honeycomb structure were obtained by vacuum-assisted impregnation of epoxy resin and high temperature curing. The chemical structures of *f*-FeNi, rGH, and rGH@FeNi were characterized and analyzed by X-ray diffraction (XRD), Raman spectroscopy, X-ray photoelectron spectroscopy (XPS) and physical property measurement system (PPMS). The microstructures of rGH@FeNi aerogel and rGH@FeNi/epoxy composites were observed by scanning electron microscope (SEM). On this basis, the effects of regular honeycomb structure and *f*-FeNi loading on electrical conductivities (*σ*), EMI SE, thermal stability and mechanical properties of rGH@FeNi/epoxy composites were detailedly investigated.

## Experimental

### Preparation of rGH@FeNi Aerogel

The GH aerogel was prepared according to our previous work [28]. The microporous Al_2_O_3_ honeycomb plate (pore size 500 μm) was firstly placed in 10 g L^−1^ GO solution prepared by oxidative exfoliation of graphite flakes [37]. Subsequently, GO was completely filled in the Al_2_O_3_ honeycomb lattice by vacuum degassing. After standing for 48 h, it was frozen in -25 °C refrigerator, and then freeze-dried for 48 h to obtain GO-Al_2_O_3_ honeycomb composite structure. Then, it was placed in dilute hydrochloric acid (HCl) to remove the Al_2_O_3_ honeycomb plate, and the GH aerogel was obtained by freeze-drying for 48 h after repeated washing with deionized (DI) water to remove the residual dilute HCl.

FeNi alloy particles (2 g) and ammonium persulfate (APS, 1 g) were added into 100 mL DI and mechanically stirred for 30 min. After stirring at 70 °C for 30 min, acrylic acid (AA, 10 mL) was added into the FeNi dispersion and stirred for 3 h. Ethylenediamine (EN, 20 mL) was added into the above mixture drop by drop and continuously stirred for 3 h. The product was washed for several times with DI water until the pH was about 7. After drying, the *f*-FeNi alloy particles were prepared.

*f*-FeNi was placed in DI water and mechanically stirred for 30 min to form a homogeneous dispersion. GH aerogel was placed in the obtained dispersion with slow stirring for 30 min, and then heated to 60 °C with continuous stirring for 2 h. GH@FeNi aerogel was obtained by freeze-drying for 48 h. The GH@FeNi aerogel was annealed under 5% hydrogen/argon (V/V) atmosphere at 600 °C for 2 h to obtain rGH@FeNi aerogel.

### Preparation of rGH@FeNi/epoxy Composites

**Fig. 1:**
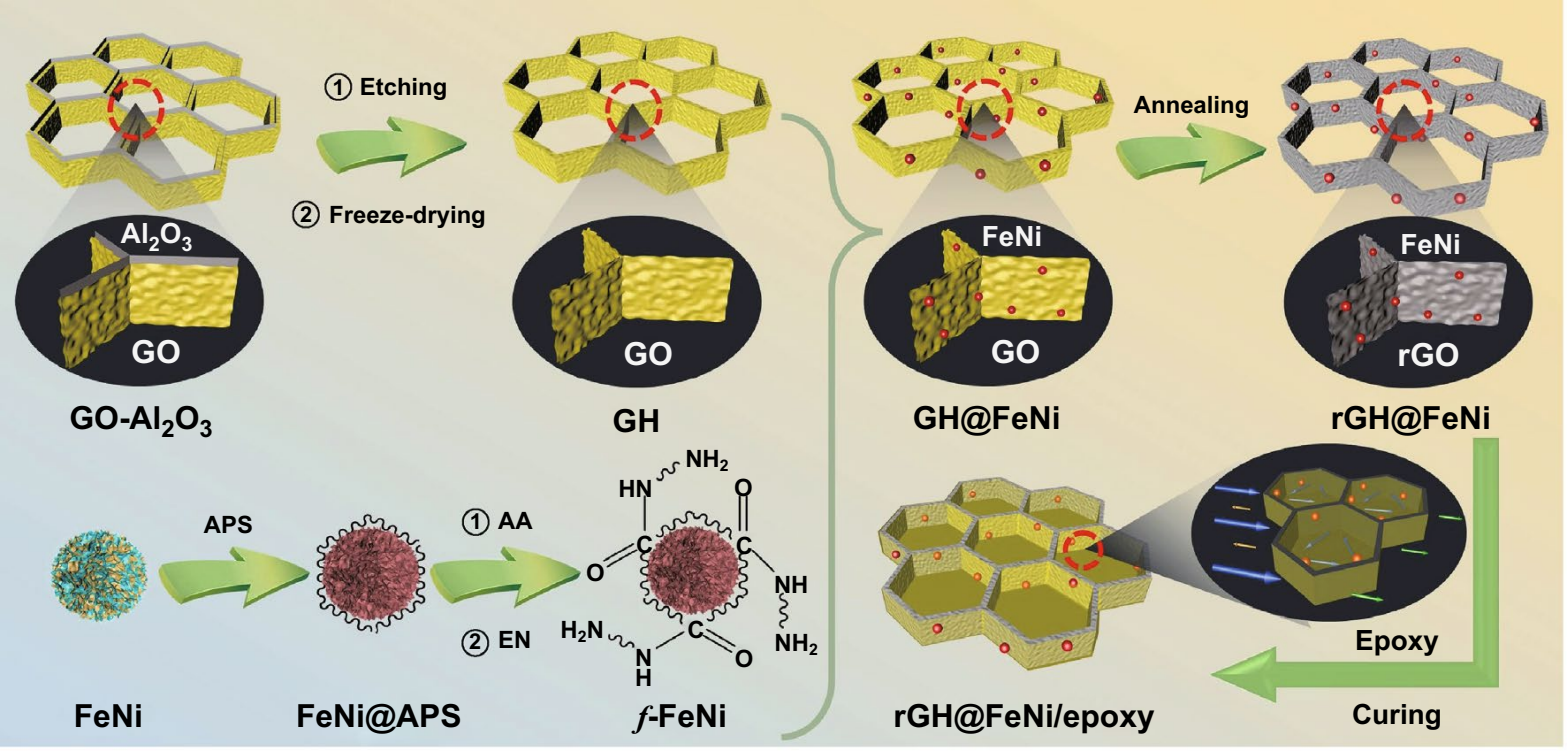
Schematic diagram of the fabrication for rGH@FeNi/epoxy composites

Epon 862 bisphenol F epoxy resin and EK3402 curing agent (100/26.5, W/W) were mixed and mechanically stirred at 70 °C for 30 min to obtain a uniform mixture. The mixture was then poured into a mold containing rGH@FeNi aerogel, followed by vacuum-assisted degassing to remove air bubbles. The rGH@FeNi/epoxy EMI shielding composites were obtained by curing at 120 °C for 5 h. Schematic diagram of the fabrication for rGH@FeNi/epoxy composites is shown in Fig. 1. The mass fractions of fillers in rGH@FeNi/epoxy composites are shown in Table 1. For comparison, rGO/FeNi/epoxy EMI shielding composites with the same rGO/FeNi mass fraction were prepared by direct blending.

## Results and Discussion

### Characterizations of rGH, *f*-FeNi, GH@FeNi and rGH@FeNi

Figure S1a shows the magnetic variation curves of rGH, *f*-FeNi and rGH@FeNi aerogels. It can be seen that *f*-FeNi shows excellent magnetic properties with saturation magnetization of 113.6 emu g^−1^, while rGH is nonmagnetic. The hysteresis loop of rGH@FeNi aerogel exhibits typical superparamagnetism [38], with a saturation magnetization of 48.7 emu g^−1^, which is slightly lower than that of *f*-FeNi. The calculated mass fraction of *f*-FeNi accounts for 43% of the total filler mass, which is consistent with the experimental design scheme. Figure S1b further shows that the rGH@FeNi aerogel has excellent magnetic properties.

**Fig. 2:**
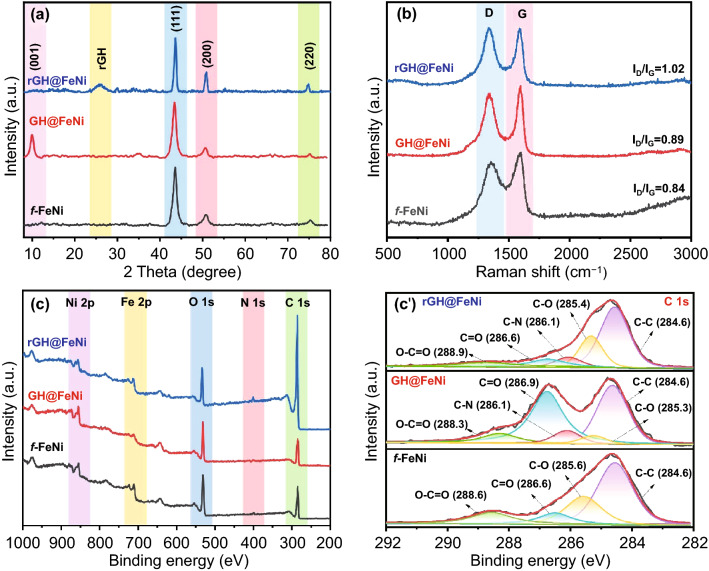
XRD (**a**), Raman (**b**), XPS spectra (**c**) and high-resolution C 1 s (**c′**) of *f*-FeNi, GH@FeNi and rGH@FeNi aerogel (rGH 1.2 wt%, *f*-FeNi 0.9 wt%)

Figure 2 is XRD (a), Raman (b) and XPS (c) spectra of *f*-FeNi, GH@FeNi and rGH@FeNi aerogels. FeNi and *f*-FeNi both present the same characteristic diffraction peak positions in XRD spectra (Fig. S2), indicating that the amino functionalized modification did not change the crystal phase structure of FeNi. The diffraction peaks at 43.6° and 50.8° correspond to (111) and (200) crystal planes of α-Fe in *f*-FeNi cubic respectively, and the diffraction peak at 5.3° corresponds to (220) crystal plane of *f*-FeNi face-centered cubic Ni [39]. In XRD spectrum of GH@FeNi aerogel, the characteristic diffraction peak at 9.9° corresponds to (001) crystal plane of GO. No obvious GO diffraction peak was observed in XRD spectrum of rGH@FeNi aerogel, but a broad and weak characteristic diffraction peak of rGO appears at 26.0°, implying that the GH has been completely reduced to rGH [40, 41]. The XRD pattern of rGH@FeNi aerogel also shows the characteristic diffraction peak of *f*-FeNi, indicating that rGH@FeNi aerogel can retain the crystal phase structure of rGO and *f*-FeNi completely. All Raman spectra show the D-band around 1343 cm^−1^ and G-band around 1589 cm^−1^. The intensity ratios of D-band and G-band (I_D_/I_G_) for *f*-FeNi, GH@FeNi and rGH@FeNi aerogels are 0.84, 0.89, and 1.02, respectively, which is because that the introduction of *f*-FeNi and reduction of GO will increase defect degree of GH. The proper defect and graphitization degree can improve the impedance matching with enhanced polarization relaxation and dipole relaxation, which is beneficial to the attenuation of electromagnetic waves [36]. All XPS spectra show the characteristic absorption peaks of C 1 s, N 1 s, and O 1 s at 284.8, 398.8, and 531.8 eV. The GH@FeNi aerogel presents characteristic absorption peaks of Fe 2p and Ni 2p at 710.8 and 854.8 eV, respectively, indicating that the *f*-FeNi is successfully grafted on the surface of GH. The C/O value of rGH@FeNi is increased significantly from 1.7 to 8.1, confirming the complete reduction of GH@FeNi [42], which is consistent with the XRD results. Figure 2c’ shows the high-resolution C 1 s spectra of *f*-FeNi, GH@FeNi and rGH@FeNi aerogel. It can be seen that the high-resolution C 1 s spectrum of *f*-FeNi presents four characteristic peaks at 284.6, 285.6, 286.6, and 288.6 eV corresponding to unoxidized carbon (C–C), carbon in hydroxyl and epoxy groups (C-O), carbon in carbonyl group (C = O), and carbon in carboxyl group (O-C = O), respectively. After grafting of *f*-FeNi on GH surface, the high-resolution C 1 s spectrum of GH@FeNi aerogel shows a characteristic peak of C-N at 286.1 eV, which is attributed to the generated C-N bond by condensation reaction between -NH_2_ on *f*-FeNi and -COOH on GH. The results demonstrate that the *f*-FeNi has been successfully grafted on the surface of GH skeleton. The peak intensity of oxygen-containing functional groups decreased significantly after thermal annealing, indicating that the GH@FeNi aerogel has been successfully reduced to rGH@FeNi aerogel [43, 44].

### Morphologies of rGH@FeNi Aerogel and rGH@FeNi/epoxy Composites

**Fig. 3:**
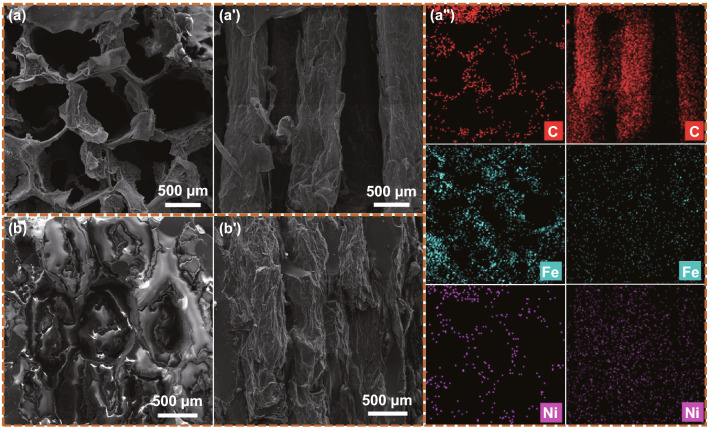
SEM images of rGH@FeNi aerogel (rGH 1.2 wt%, *f*-FeNi 0.9 wt%) along the cross and longitudinal sections (**a & a′**), elemental mapping images of C, Fe and Ni for rGH@FeNi aerogel (**a′′**), SEM images of rGH@FeNi/epoxy composites (rGH 1.2 wt%, *f*-FeNi 0.9 wt%) along the cross and longitudinal sections (**b & b′**)

Figure 3 shows SEM images of rGH@FeNi aerogel and rGH@FeNi/epoxy composites. As can be seen from Fig. 3a, a′, the rGH@FeNi aerogel presents anisotropic structures with highly regular hexagonal honeycomb structure in cross section and highly oriented prismatic structure in longitudinal sections. The side length of honeycomb cells is about 500 μm. When the Al_2_O_3_ template is immersed in GO solution, the surface of Al_2_O_3_ template is ionized with released Al^3+^ spontaneously diffused into the weakly acidic GO solution. This significantly increases the ionic strength near Al_2_O_3_ template and causes the negatively charged GO nanosheets to aggregate on the Al_2_O_3_ template and form GO hydrogel with hollow hexagonal honeycomb structure. The distribution of C, Fe, and Ni elements in EDS diagram (Fig. 3a′′) also shows that the *f*-FeNi has been successfully grafted on the surface of rGH skeleton, and the rGH@FeNi aerogel present hexagonal honeycomb and prismatic structures in cross and longitudinal sections, respectively. Therefore, the *f*-FeNi and rGO construct a more effective 3D conductive and magnetic path. As shown in Fig. 3b, b′, the rGH@FeNi aerogel can maintain its morphology without obvious damage after the back-filling of epoxy resin. The fracture surfaces of rGH@FeNi/epoxy composite still present the original regular hexagonal honeycomb and prismatic structures in cross and longitudinal sections, respectively. The construction of highly oriented hexagonal honeycomb structure is of great significance for the improvement of σ, EMI shielding and mechanical properties of the composites.

### Electrical Conductivities and EMI Shielding Performances of rGH@FeNi/Epoxy Composites

**Fig. 4:**
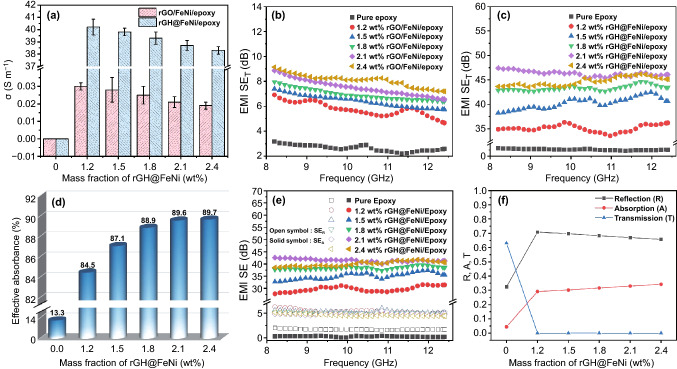
*σ* of rGO/FeNi/epoxy and rGH@FeNi/epoxy composites (**a**), EMI SE_T_ of rGO/FeNi/epoxy composites (**b**), EMI SE_T_ (**c**), effective absorbance (**d**), EMI SE_A_ & SE_R_ (**e**) and R, A and T coefficients (**f**) of rGH@FeNi/epoxy composites

Figure 4a is *σ* of rGO/FeNi/epoxy and rGH@FeNi/epoxy EMI shielding composites. With the same mass fraction of rGO and rGH (1.2 wt%), the two kinds of composites both exhibit decreased *σ* as the *f*-FeNi mass fraction increases. When the mass fraction of rGH@FeNi is 2.1 wt% (the mass fraction of *f*-FeNi is 0.9 wt%), the *σ* of rGH@FeNi/epoxy composites is 38.7 S m^−1^, which is much higher than that of rGO/FeNi/epoxy composites with the same mass fraction. However, it is slightly lower than the *σ* (40.2 S m^−1^) of rGH/epoxy composites without *f*-FeNi. For rGO/FeNi/epoxy composites, the rGO tends to aggregate easily in the epoxy resin matrix during physical blending due to its high surface energy, resulting in the difficulty of forming perfect conductive path and relatively low *σ*. When rGO is constructed into rGH with the assistance of honeycomb template, the rGO forms an efficient 3D continuous conductive network inside the epoxy resin matrix, thus leading to the greatly increased *σ* [27, 45]. As the mass fraction of magnetic *f*-FeNi increases, the contact resistance between rGH conductive networks gradually increases due to the intrinsic electrical insulation of *f*-FeNi, leading to the reduced *σ* of rGH@FeNi/epoxy composites to a certain extent.

Figure 4b, c shows the EMI SE_T_ values of rGO/FeNi/epoxy and rGH@FeNi/epoxy EMI shielding composites, respectively. With the same mass fraction of rGO and rGH (1.2 wt%), the EMI SE_T_ of rGO/FeNi/epoxy composites gradually increases as the *f*-FeNi mass fraction increases, while the that of rGH@FeNi/epoxy composites firstly increases and then decreases. When the mass fraction of rGH@FeNi is 2.1 wt% (the mass fraction of *f*-FeNi is 0.9 wt%), the EMI SE_T_ of rGH@FeNi/epoxy composites reach the maximum value of 46 dB, which is 5.8 times of that of rGO/FeNi/epoxy composites (8 dB) with same mass fraction. On the one hand, the honeycomb structure in rGH@FeNi/epoxy composites constructs a highly effective 3D conductive rGH network inside the epoxy resin matrix, leading to the significantly increased *σ* and thus improved ohmic loss of incident electromagnetic waves [46, 47]. On the other hand, the continuous 3D conductive/magnetic rGH network can enhance the multiple reflection and scattering of electromagnetic waves in rGH@FeNi/epoxy composites and extend the transmission path of electromagnetic waves, which contribute to the attenuation of electromagnetic waves and EMI SE_T_ of the composites [48, 49]. Meanwhile, the increasing mass fraction of *f*-FeNi can enhance the hysteresis loss of electromagnetic waves in the rGH@FeNi/epoxy composites. Nevertheless, as the mass fraction of *f*-FeNi further increases to 1.2 wt%, the excessive *f*-FeNi is detrimental to the gelation of rGH and brings damage to the construction of rGH@FeNi continuous network to some extent (Fig. S3). Therefore, the EMI SE_T_ of rGH@FeNi/epoxy composites decreases slightly when the mass fraction of rGH@FeNi is 2.4 wt%.

Figure 4d is the electromagnetic wave effective absorbance of rGH@FeNi/epoxy EMI shielding composites (calculated by Eq. S9). It can be seen that with a constant mass fraction of rGH (1.2 wt%), the electromagnetic wave effective absorbance of rGH@FeNi/epoxy composites gradually increases as the mass fraction of *f*-FeNi increases. When the mass fraction of rGH@FeNi is 2.1 wt% (the mass fraction of *f*-FeNi is 0.9 wt%), the electromagnetic wave absorption rate of rGH@FeNi/epoxy composites is 89.6%, which is higher than that of rGH/epoxy composite without *f*-FeNi (84.5%). This is because that the loading of *f*-FeNi enhances the hysteresis loss of rGH@FeNi/epoxy composites to electromagnetic waves due to the improved impedance matching between rGH 3D conductive skeleton and epoxy resin matrix, thus leading to the significantly increased effective absorbance of the composites [36, 39].

Figure 4e shows the absorpted shielding effectiveness (SE_A_) and reflected shielding effectiveness (SE_R_) of rGH@FeNi/epoxy EMI shielding composites in X-band. As can be seen, with the increase of *f*-FeNi mass fraction, the SE_R_ of rGH@FeNi/epoxy composites remain at about 5 dB with the constant rGH mass fraction (1.2 wt%). The SE_A_ and SE_T_ show the same variation tendency, both of which firstly increase and then decrease with the increase of *f*-FeNi mass fraction. When the mass fraction of rGH@FeNi is 2.1 wt% (the mass fraction of *f*-FeNi is 0.9 wt%), the SE_A_ of rGH@FeNi/epoxy composites can reach 41 dB. This is mainly attributed to the fact that the incorporation of *f*-FeNi can improve the impedance matching of composites and enhance the hysteresis loss of electromagnetic waves in composites, thus improving the SE_A_ value. For practical application, the EMI shielding mechanism of rGH@FeNi/epoxy composites is evaluated by reflection (R), absorption (A) and transmission (T) coefficients (Eqs. S3-S5). As shown in Fig. 4f, the rGH@FeNi/epoxy composites with various *f*-FeNi mass fraction all show extremely low T coefficient. When the mass fraction of rGH@FeNi is 2.1 wt% (the mass fraction of *f*-FeNi is 0.9 wt%), the T coefficient of rGH@FeNi/epoxy EMI shielding composites is as low as only 2 × 10^–5^, demonstrating that the incorporation of *f*-FeNi can significantly improve the EMI shielding performances. At the same time, the R value of rGH@FeNi/epoxy composites gradually decreases with the constant rGH mass fraction (1.2 wt%) and increased *f*-FeNi mass fraction. It is because that the incorporated *f*-FeNi can reduce *σ* and increase the magnetism, which can improve the impedance matching between rGH@FeNi cellular network and external space. This results in the increased absorption and reduced reflection of electromagnetic waves on the surface of rGH@FeNi/epoxy composites, and is beneficial to reducing the secondary pollution of electromagnetic waves [50, 51].

**Fig. 5:**
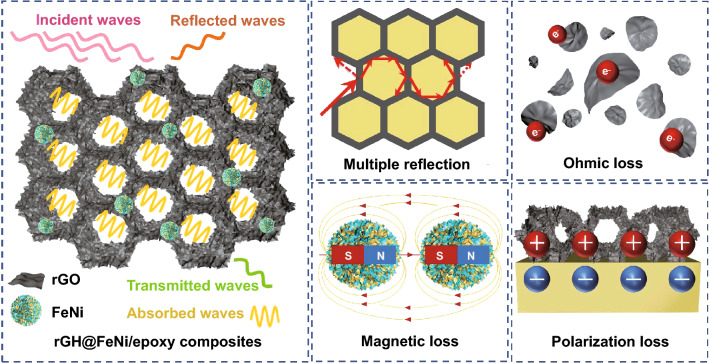
EMI shielding mechanism of rGH@FeNi/epoxy composites

When the incident electromagnetic wave reaches the surface of rGH@FeNi/epoxy EMI shielding composites, some incident electromagnetic waves are reflected on the surface of rGH@FeNi/epoxy composites due to the impedance mismatch between external space and composites, while the rest electromagnetic waves enter the interior of rGH@FeNi/epoxy composites. First of all, when electromagnetic waves pass through the honeycomb wall of rGH, the highly conductive rGH will generate induced current and cause ohmic losses of electromagnetic waves [22, 52]. Secondly, the existence of the honeycomb conductive/magnetic structure lengthens the transmission path of electromagnetic waves in the composites, leading to the enhanced attenuation of electromagnetic waves by multiple reflection and scattering [53]. Meanwhile, the hysteresis loss caused by the magnetic *f*-FeNi also contribute to the absorption of incident electromagnetic waves [54]. In addition, as the σ of rGH is much higher than that of epoxy resin, there are different σ values on the two sides of interfaces. Under the external electric field, the positive charges and negative charges accumulate in the rGH and epoxy resin sides respectively, thus resulting in the interface polarization loss [55, 56]. Therefore, the multiple loss mechanism composed of ohmic loss, internal multiple reflections, hysteresis loss and interface polarization loss greatly improve the EMI shielding performance of rGH@FeNi/epoxy composites. The EMI shielding mechanism of rGH@FeNi/epoxy composites is shown in Fig. 5.

### Thermal Stabilities of the rGH@FeNi/epoxy Composites

**Fig. 6:**
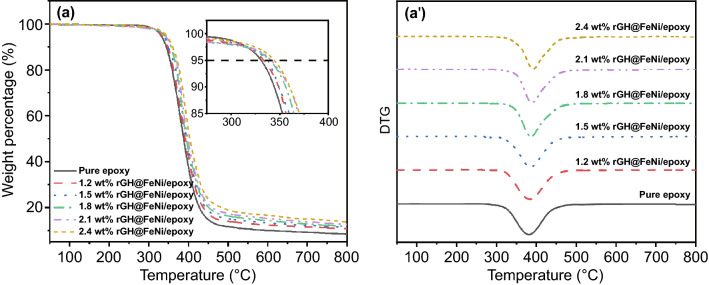
TGA (**a**) and DTG (**a′**) curves of the rGH@FeNi/epoxy composites

Figure 6a, a′ show the thermal gravimetric analysis (TGA) and derivative thermogravimetric (DTG) curves of pure epoxy and rGH@FeNi/epoxy EMI shielding composites, respectively [57], with the corresponding characteristic data shown in Table 2. The heat-resistance index (*T*_HRI_) is calculated by Eq. 1 to evaluate the long-term service temperature of polymer nanocomposites.

1 $$T_{Heat - resis\tan ce \, index} = \, 0.49^{\prime}\left[ {T_{5} + 0.6^{\prime}\left( {T_{30} - T_{5} } \right)} \right]$$

where *T*_5_ and *T*_30_ correspond to the decomposition temperatures of 5% and 30% weight loss, respectively. It can be seen that the heat-resistance index (*T*_HRI_) and temperature at the maximum decomposition rate (*T*_dmax_) of pure epoxy are 173.5 °C and 381.3 °C, respectively. The *T*_5_, *T*_30_, *T*_HRI_, and *T*_dmax_ of rGH@FeNi/epoxy composites are significantly improved with the increased FeNi mass fraction and constant rGH mass fraction (1.2 wt%). When the mass fraction of rGH@FeNi is 2.1 wt% (the mass fraction of FeNi is 0.9 wt%), the *T*_HRI_ and *T*_dmax_ of rGH@FeNi/epoxy composites can reach 179.1 and 389.0 °C, respectively, which are 5.6 and 7.7 °C higher than those of pure epoxy, respectively. This is because that the incorporation of *f*-FeNi with excellent thermal stability can improve the heat resistance of rGH@FeNi/epoxy composites. In addition, the 3D honeycomb network structure formed by rGH@FeNi can effectively prevent the thermal degradation of rGH@FeNi/epoxy composites, slow down the thermal degradation rate, thus resulting the improved thermal stability [58, 59].

**Table 1:** Filler mass fraction of rGH@FeNi/epoxy composites

Samples	rGH@FeNi (wt%)	rGH (wt%)	*f*-FeNi alloy (wt%)
Pure epoxy	0	0	0
1.2 wt% rGH@FeNi/epoxy	1.2	1.2	0
1.5 wt% rGH@FeNi/epoxy	1.5	1.2	0.3
1.8 wt% rGH@FeNi/epoxy	1.8	1.2	0.6
2.1 wt% rGH@FeNi/epoxy	2.1	1.2	0.9
2.4 wt% rGH@FeNi/epoxy	2.4	1.2	1.2

**Table 2:** Thermal characteristic data of the rGH@FeNi/epoxy composites

Sample	Weight loss temperature (°C)		*T*_HRI_ (°C)	*T*_dmax_ (°C)
	*T*_5_	*T*_30_		
Pure epoxy	330.8	369.7	173.5	381.3
1.2 wt% rGH@FeNi/epoxy	333.7	373.3	175.2	383.2
1.5 wt% rGH@FeNi/epoxy	337.3	377.3	177.0	385.0
1.8 wt% rGH@FeNi/epoxy	340.0	379.0	178.1	386.7
2.1 wt% rGH@FeNi/epoxy	341.3	381.7	179.1	389.0
2.4 wt% rGH@FeNi/epoxy	344.3	386.7	181.2	392.3

### Dynamic Mechanical Analysis of the rGH@FeNi/epoxy Composites

**Fig. 7:**
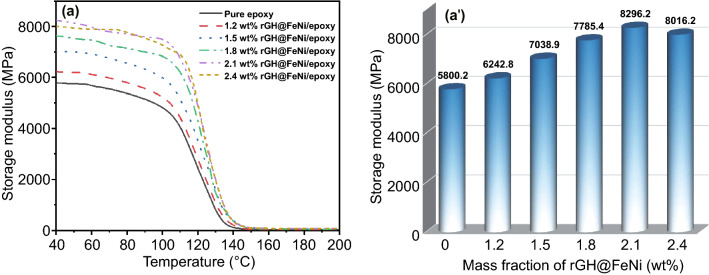
DMA curves (**a**) and *E*_s_ (**a′**) of the rGH@FeNi/epoxy composites

Figure 7a, a′ shows the dynamic mechanical analysis (DMA) curves of rGH@FeNi/epoxy EMI shielding composites and their corresponding storage modulus (*E*_s_) at the temperature of 40 °C, respectively. It can be seen that the *E*_s_ of pure epoxy is 5800.2 MPa. With the constant mass fraction of rGH (1.2 wt%), the *E*_s_ of rGH@FeNi/epoxy composites first increase and then decrease with the increasing *f*-FeNi mass fraction. When the mass fraction of rGH@FeNi is 2.1 wt% (the mass fraction of *f*-FeNi is 0.9 wt%), the rGH@FeNi/epoxy composites exhibit the largest *E*_s_ of 8296.2 MPa, which is 43% higher than that of pure epoxy. The related reason is that in incorporation of *f*-FeNi increases the surface roughness of rGH@FeNi aerogel, which can inhibit the activity of molecular chain segments of epoxy resin due to the interface binding effect, thus leading to the improved *E*_s_ [60, 61]. With the further increase of *f*-FeNi mass fraction (2.4 wt%), the excessive FeNi destroys the 3D honeycomb network structure of rGH@FeNi aerogels to some extent, resulting in the slight decrease of *E*_s_.

## Conclusions

In conclusion, the rGH@FeNi/epoxy EMI shielding composites with regular 3D honeycomb structures were prepared by sacrificial template, freeze-drying and vacuum-assisted impregnation of epoxy resin. Benefitting from the construction of 3D honeycomb structure and electromagnetic synergistic effect, the rGH@FeNi/epoxy composites with a low rGH@FeNi mass fraction of 2.1 wt% (rGH and *f*-FeNi are 1.2 and 0.9 wt% respectively) exhibit a high EMI shielding effectiveness (EMI SE) of 46 dB, which is 5.8 times of that (8 dB) for rGO/FeNi/epoxy composites with the same rGO/FeNi mass fraction. Meanwhile, the rGH@FeNi/epoxy composites also possess excellent thermal stability (*T*_HRI_ and *T*_dmax_ were 179.1 and 389.0 °C, respectively) and mechanical properties (*E*_s_ was 8296.2 MPa). It is believed that the epoxy-based EMI shielding composites with comprehensive performances have great application potentials in consumer electronics, artificial intelligence and information security, etc.

## Supplementary Information

Below is the link to the electronic supplementary material.

Supplementary file1 (PDF 296 KB)
