# Supplementary material for: High-Efficiency Electromagnetic Interference Shielding of rGO@FeNi/Epoxy Composites with Regular Honeycomb Structures
Source: Nanomicro Lett. 2022 Jan 27;14:51. doi: 10.1007/s40820-022-00798-5 (PMC8795265; doi:10.1007/s40820-022-00798-5)
Supplement: Supplementary file 1 — Supplementary file1 (PDF 296 KB) [file 40820_2022_798_MOESM1_ESM.pdf]

Supporting Information for

High-Efficiency Electromagnetic Interference Shielding of rGO@FeNi/Epoxy Composites with Regular Honeycomb Structures

Ping Song[1], Zhonglei Ma[1, *], Hua Qiu[1], Yifan Ru[2], Junwei Gu[1, *]

[1]Shaanxi Key Laboratory of Macromolecular Science and Technology, School of Chemistry and Chemical Engineering, Northwestern Polytechnical University, Xi'an 710072, China

[2]Queen Mary University of London Engineering School, Northwestern Polytechnical University, Xi'an 710072, P. R. China.

*Corresponding authors. E-mail: mazl@nwpu.edu.cn (Z. Ma); gjw@nwpu.edu.cn or nwpugjw@163.com (J. Gu)

S1 Experimental Section

S1.1 Main Materials

Bisphenol F epoxy (Epon 862) was supplied by Hexion Inc (Columbus, USA). Iron nickel alloy powder (Ni: 30%) was provided by Chengdu unclear 857 new material Co. Ltd (Sichuan, China). Graphite flake (325 mesh, 99.8%) was purchased from Alfa Aesar Co. Ltd. (Shanghai China). Al_2O_3 honeycomb plates (22.86×10.16×3.00 mm^3, pore size 500 μm) were obtained from Suzhou Beecore Honeycomb Materials Co. Ltd (Jiangsu, China). Potassium persulfate ($K_2S_2O_8$, ⩾99%), phosphorus pentoxide (P_2O_5, ≥98%), potassium permanganate ($KMnO_4$, ⩾99%), sulfuric acid (H_2SO_4, 98%), hydrogen peroxide (H_2O_2, 30 wt%), hydrochloric acid (HCl, 37 wt%), ammonium persulfate (APS, $NH(NH_4)_2S_2O_8$, ≥99%), ethylenediamine (EN, $C_2H_8N_2$, ≥99%) and acrylic acid (AA, $C_3H_4O_2$, ≥99%) were all purchased from Beijing Chemical Factory (Beijing, China). All water used was deionized (DI) water.

S1.2 Characterizations

X-ray diffraction (XRD) patterns of the samples were obtained on a Shimadzu-7000 type X-ray diffraction (Shimadzu, Japan, λ=0.154 nm). Raman spectroscopies were measured on a WITec Alpha300R (PE Corp., England) with a He-Ne laser, tuned at 532 nm. X-ray photoelectron spectroscopy (XPS) spectra of the samples were carried out using a PHI5400 equipment (PE Corp., England). Thermogravimetric analysis (TGA) of the samples were carried out under air atmosphere using STA 449F3 (NETZSCH C Corp., Germany) over the temperature ranging from 40 to 800 ^{o}C with a heating rate of 10 ^{o}C/min. Dynamic mechanical analysis (DMA) of the samples were determined by DMA/SDTA861e (Mettler Toledo Corp., Switzerland) with a frequency of 1 Hz and a heating rate of 5°C/min in the temperature range from 40 to 200 ^{o}C, with the sample dimension of 55×10×4 mm^3. Morphologies of the samples were observed by scanning electron microscope (SEM, VEGA3-LMH, ESCAN Corp., Czech Republic). Magnetic properties of the samples were investigated by CFMS-14T physical property measurement system (PPMS) (Cryogenic, England). Direct current (DC) electrical conductivity of the samples was measured by a four-probe method at room temperature, and the corresponding dimension was 22.86×10.16×3.00 mm^3. EMI shielding parameters of the

samples were tested by vector network analyzer (VNA, MS4644A, Anritsu) using wave-guide method in X-band (8.2-12.4 GHz) according to ASTM D5568-08, and the corresponding dimension was 22.86×10.16×3.00 mm^3. The incident direction of electromagnetic waves was perpendicular to honeycomb pore for EMI SE measuring.

S2 Magnetism of rGH, f-FeNi and rGH@FeNi

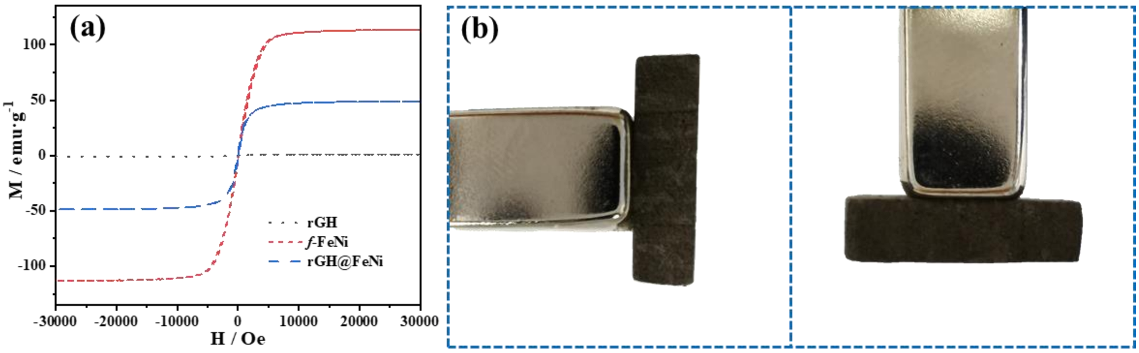

Fig. S1 Magnetization curves of rGH, *f*-FeNi and rGH@FeNi (rGH 1.2 wt%, *f*-FeNi 0.9 wt%) (**a**); Illustration for the magnetism of rGH@FeNi/epoxy composites (**b**)

S3 Characterization of FeNi Alloy

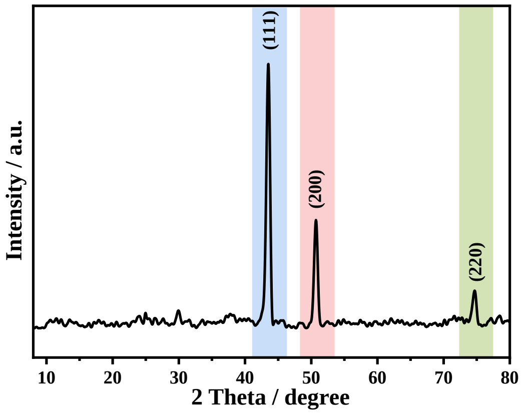

Fig. S2 XRD spectra of FeNi alloy

S4 Characterization of rGH@FeNi/epoxy Composites

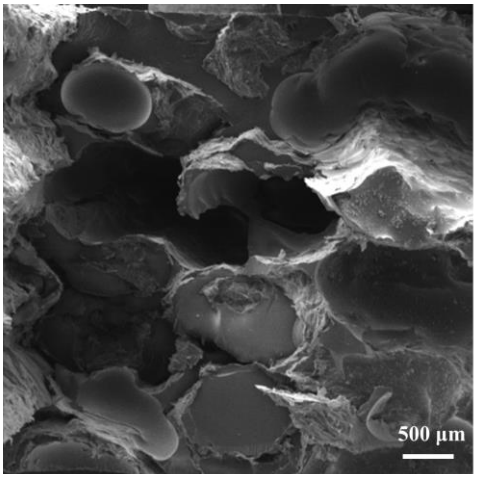

Fig. S3 SEM image of rGH@FeNi/epoxy composites (mass fraction of rGH@FeNi is 2.4 wt%)

S5 EMI SE: Theory and Measurements

EMI SE (unit: dB) is an important indicator to evaluate the EMI shielding performances of EMI shielding materials. It is defined as the attenuation value of electromagnetic waves for EMI shielding materials, and can be expressed as follows:

$$SE=20\lg\left(\frac{E_0}{E_s}\right)=20\lg\left(\frac{H_0}{H_s}\right)=20\lg\left(\frac{P_0}{P_s}\right) \tag{S1}$$

In **Eq. S1**, E_0, H_0 and P_0 represent the intensities of electric field, magnetic field and power before EMI shielding respectively, while E_s, H_s and P_s represent the intensities of electric field, magnetic field and power after EMI shielding respectively.

Currently, the Schelkunoff formula is usually used to calculate the EMI SE. Based on the transmission line theory, the formula considers that the total EMI shielding efficiency (SE_T) is the sum of reflection loss (SE_R), absorption loss (SE_A) and multiple reflection loss (SE_M) of electromagnetic waves in materials, which can be expressed as:

$$SE_T=SE_R+SE_A+SE_M \tag{S2}$$

In **Eq. S2**, SE_R, SE_A and SE_T can be calculated by the following equations:

$$T=S_{12}^2=S_{21}^2 \tag{S3}$$

$$R=S_{11}^2 \tag{S4}$$

$$A=1-T-R \tag{S5}$$

$$SE_T(dB) = -10\log(T) \tag{S6}$$

$$SE_R(dB) = -10\log(1-R) \tag{S7}$$

$$SE_A(dB) = -10\log\left(\frac{T}{1-R}\right) \tag{S8}$$

where S_{11} represents the reflection coefficient, S_{12} represents the transmission coefficient, S_{21} represents the reverse transmission coefficient, and S_{22} represents the reverse reflection coefficient. The four coefficients are obtained by VNA. The electromagnetic wave effective absorbance represents the proportion of absorption loss in the total EMI SE, and can be expressed as:

$$\text{Effective absorbance}=\frac{SE_A}{SE_T}\times 100\% \tag{S9}$$
